# Trans-tail regulation-mediated suppression of cryptic transcription

**DOI:** 10.1038/s12276-021-00711-x

**Published:** 2021-11-29

**Authors:** Jungmin Choi, Zae Young Ryoo, Dong-Hyung Cho, Hyun-Shik Lee, Hong-Yeoul Ryu

**Affiliations:** 1 Department of Biomedical Sciences, Korea University College of Medicine, Seoul, 02841 Republic of Korea; 2 BK21 FOUR KNU Creative BioResearch Group, School of Life Sciences, College of National Sciences, Kyungpook National University, Daegu, 41566 Republic of Korea

**Keywords:** Histone post-translational modifications, Epigenetics, Epigenetics

## Abstract

Crosstalk between post-translational modifications of histone proteins influences the regulation of chromatin structure and gene expression. Among such crosstalk pathways, the best-characterized example is H2B monoubiquitination-mediated H3K4 and H3K79 methylation, which is referred to as trans-tail regulation. Although many studies have investigated the fragmentary effects of this pathway on silencing and transcription, its ultimate contribution to transcriptional control has remained unclear. Recent advances in molecular techniques and genomics have, however, revealed that the trans-tail crosstalk is linked to a more diverse cascade of histone modifications and has various functions in cotranscriptional processes. Furthermore, H2B monoubiquitination sequentially facilitates H3K4 dimethylation and histone sumoylation, thereby providing a binding platform for recruiting Set3 complex proteins, including two histone deacetylases, to restrict cryptic transcription from gene bodies. The removal of both ubiquitin and SUMO, small ubiquitin-like modifier, modifications from histones also facilitates a change in the phosphorylation pattern of the RNA polymerase II C-terminal domain that is required for subsequent transcriptional elongation. Therefore, this review describes recent findings regarding trans-tail regulation-driven processes to elaborate on their contribution to maintaining transcriptional fidelity.

## Cell biology: Interaction of DNA-winding proteins ensures correct gene expression

Crosstalk between different DNA-winding proteins, or histones, is a mechanism of molecular fidelity that helps prevent the initiation of aberrant gene expression, which may contribute to cancer and neurodegenerative disease. A team from South Korea, led by Jungmin Choi from the Korea University College of Medicine in Seoul and Hong-Yeoul Ryu from Kyungpook National University in Daegu, review the ways in which different histone proteins chemically modify parts of each other’s structure to regulate their functions. These modifications affect how histones interact with DNA, which in turn alters the dynamics of other factors implicated in gene expression. The correct interaction of histones is necessary to prevent the gene expression machinery from starting RNA synthesis from the wrong sites. Accurate control of these mechanisms is essential for cellular wellbeing

## Introduction

In eukaryotic organisms, nuclear DNA is compressed into a high-order packaged structure referred to as chromatin, which comprises repeating building blocks called nucleosomes^1^. Each nucleosome is made up of ~147 bp of DNA wrapped around an octamer of histone proteins containing two copies of each of histones H2A, H2B, H3, and H4. Subsequently, histones can then undergo several types of post-translational modifications, including methylation at arginine (R), phosphorylation at serine (S) and threonine (T), and other diverse types of modifications (acetylation, methylation, ubiquitylation, sumoylation, biotinylation, and ADP-ribosylation) at the lysine (K) region^2,3^. However, studies have shown that these modifications alter interactions between DNA and histones, thereby allowing the recruitment of chromatin-modifying enzymes and transcription factors^4^.

Histone modifications can also modulate the establishment of other modifications within the same histone (in *cis*) or in a different histone (in *trans*), thereby providing crosstalk among the histones^5^. Such crosstalk generates complex signals that facilitate or repress chromatin-mediated processes. For instance, in humans, phosphorylation at H3S10 and acetylation at H4K16 act cooperatively to recruit the P-TEFb (positive transcriptional elongation factor b) to the nucleosome, thus promoting transcriptional elongation^6^. Additionally, Chk1-mediated H3T11 phosphorylation allows the acetylation of H3K14 by Gcn5 for the transcriptional activation of genes encoding products involved in cell cycle regulation. However, DNA damage drives this crosstalk in the reverse direction to induce transcriptional repression^7^. A study further showed that the yeast SAGA (Spt–Ada–Gcn5–acetyltransferase) complex stimulated acetylation on nucleosomes containing methylated H3K4 via the tandem Tudor domains of the Sgf29 subunit^8^, and this histone acetylation conversely provoked Set1-driven H3K4 methylation^9^.

**Fig. 1:**
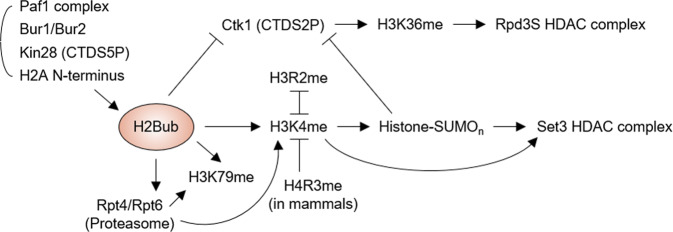
H2B monoubiquitination-centered crosstalk pathway in S. cerevisiae. The Paf1 complex, Bur1/Bur2 and Kin28 kinases, and N-terminal tails of H2A facilitate H2B monoubiquitination (ub) during yeast transcription. H2Bub then promotes H3K4 and H3K79 methylation (me) both directly and via the proteasomal ATPases Rpt4 and Rpt6. H3R2me mutually antagonizes H3K4me, whereas H4R3me interferes with the binding ability of H3K4 methyltransferase (no reports of H4R3me in *S. cerevisiae*). Although either H2Bub- or H3K4me-stimulated histone (poly)sumoylation blocks the recruitment of Ctk1 CTDS2 kinase, facilitating H3K36me modification for loading of the Rpd3S HDAC complex, both H3K4me and histone (poly)sumoylation are required for the chromatin binding of the Set3 HDAC complex.

The best-characterized histone crosstalk occurs between H2B monoubiquitination (ub)-dependent H3K4 and K79 methylation, which is an evolutionarily conserved trans-tail pathway, to maintain dynamic chromatin structure during transcription^10,11^. Recent reports have revealed that this traditional crosstalk is regulated in a more complex manner and is involved in more diverse functions than others have reported (Fig. 1). A study showed that H2BK123 ub-stimulated H3K4 di-(me2), but not tri-(me3) methylation promoted histone sumoylation, thereby providing a binding site for the Set3 HDAC (histone deacetylase) complex^12^. This Set3 complex-mediated histone deacetylation in the 5′ ORF region was then reported to facilitate the suppression of spurious transcriptional initiation in genes^13,14^. On the basis of these presented facts, this review provides an overview of newly discovered functions of the trans-histone H3K4 methylation process regulated by H2Bub in restricting cryptic transcription.

### Intragenic cryptic transcription

**Fig. 2:**
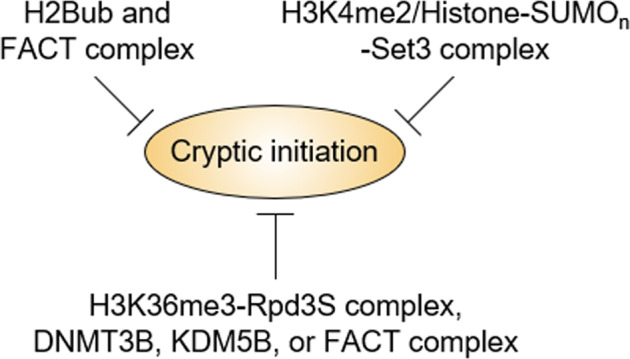
Epigenetic pathways inhibiting cryptic transcription. Cryptic transcriptional initiation within the ORF is suppressed by cooperation between H2Bub and the FACT complex, H3K4me2 and the histone (poly)sumoylation-mediated Set3 HDAC complex, and the H3K36me3-dependent association of the Rpd3S complex, DNMT3B, KDM5B, or FACT complex.

Transcription is a complex process that requires the sequential assembly of many factors, including chromatin-modifying and remodeling enzymes that act to elongate the RNA polymerase machinery on nucleosomal templates^15,16^. During transcription, the chromatin structure must be dynamically modified and reorganized, and the failure of its reassembly often leads to the exposure of cryptic promoter elements that initiate aberrant transcription from intragenic regions in a TATA-dependent or TATA-independent manner^17^. One such example is that cryptic transcripts are considerably increased in mutants of yeast histone chaperones Spt6, Spt16 (a subunit of the FACT complex), or Rtt106, which maintain nucleosome occupancy and DNA accessibility^18–22^. Moreover, following transcriptional elongation, histone acetylation should be erased to prevent cryptic transcription within the ORF region (Fig. 2). Yeast Rpd3 is the HDAC enzyme with the best-established role in inhibiting cryptic transcriptional initiation. Furthermore, Set2 histone methyltransferase-mediated H3K36me3 acts as an epigenetic mark for Rpd3S complex loading, resulting in the histone deacetylation of the ORF^23–25^. According to previous studies in *S. cerevisiae*, this Set2-Rpd3S pathway governs cryptic initiation in ~30% of yeast genes, suggesting that H3K36me3-originated chromatin modifications are important for maintaining genome integrity^26,27^. In mammals, H3K36me3 on gene bodies also acts as a repressor of aberrant intragenic transcription by promoting the recruitment of DNMT3B DNA methyltransferase, KDM5B H3K4 demethylase, or the FACT complex^28–31^. The mechanisms blocking cryptic initiation in yeast share some similarities with those in mammals.

### H2B monoubiquitination

Eukaryotic histones H2A and H2B are apparent targets of monoubiquitination, although H2A modification has not been detected in yeast^32^. Whereas a single ubiquitin moiety has been reported to be conjugated to K119 of H2A and K120 of H2B in mammals^32^, the site of H2Bub corresponds to K123 in *S. cerevisiae*^33^, K119 in *Schizosaccharomyces pombe*^34^ and K143 in *Arabidopsis*^35^. However, *S. cerevisiae* is a preferred model for studying the role and mechanism of H2Bub, and such studies have been extended to higher eukaryotes. Furthermore, the E2 conjugation enzymes Rad6 and the E3 ligase Bre1 mediate the H2Bub process, and these enzymes are preferentially enriched across transcribed regions and correlate with transcriptional processes^36^. This modification is also dynamically regulated by two ubiquitin hydrolases, Ubp8 and Ubp10, which appear to regulate distinct chromatin regions^37^.

During the transcription cycle, diverse factors dynamically regulate the level of H2Bub, which also facilitates transcription activation. At the stage of transcriptional initiation, the Paf1 complex influences the interaction of Rad6 and Bre1 with RNA polymerase II (RNAPII) and subsequently favors the H2Bub step; however, the Paf1 complex does not affect the localization of Rad6 and Bre1 in the promoter region^38,39^. Rad6 localization is not affected because mutations impair the association of Rad6 with elongating RNAPII in the Rtf1 subunit of the Paf1 complex. Therefore, the Paf1 complex is proposed to be required for Rad6 and Bre1 progression during transcriptional elongation^38^. Other studies have shown that the Bur1/Bur2 kinase complex also affects the level of H2Bub by regulating Paf1 complex recruitment and Rad6 phosphorylation^40,41^.

In particular, H2Bub is closely linked to the C-terminal domain (CTD) of the largest subunit of RNAPII, which is sequentially subjected to phosphorylation at S2 and S5^42^. There, while the loss of Kin28 (CDK7 in mammals), which ensures phosphorylation at CTDS5, inhibits H2Bub^38^, the removal of ubiquitin from H2B by Ubp8 allows the recruitment of Ctk1 CTDS2 kinase (P-TEFb/CDK9 in mammals) to promote the transition between the initiation and elongation steps. Hence, subsequent H3K36 methylation for binding of Rpd3S HDAC complex to gene bodies is favored^25,43^. Additionally, H2Bub functions cooperatively with the histone chaperone FACT complex to inhibit spurious transcriptional initiation^44,45^ (Fig. 2). Therefore, such dynamic H2B ubiquitination is critical for efficient transcriptional elongation.

### Trans-tail regulation

Previous studies have demonstrated that H2Bub unidirectionally facilitates the methylation of K4 and K79 at trans-histone H3, which is catalyzed by methyltransferases Set1 and Dot1 in *S. cerevisiae*, respectively^36^. The loss of H2Bub in *rad6Δ*, *bre1Δ* or the arginine substitution mutant of H2BK123 (H2BK123R) results in the abolition of H3K4me3/me2 and H3K79me3 along with reductions in H3K4 monomethylation (me1) and H3K79me2^46,47^, while increased levels of H2Bub resulting from the loss of deubiquitinases Ubp8 or Ubp10 cause increases in methylation of H3K4 and H3K79^48–50^. However, some groups suggest that the absence of H2B ubiquitination is insufficient to completely interrupt further trans-tail H3 methylation^51,52^ or that crosstalk between H2Bub and Dot1 is bidirectional in a methyltransferase activity-independent manner^53^.

Typically, two models have been proposed to explain the trans-tail regulation pathway: the wedge and bridge models^54^. Since ubiquitination is a bulky post-translational modification, H2Bub is predicted to act as a “wedge” that opens chromatin locally, thereby allowing the access of chromatin-modifying enzymes, including histone methyltransferases^54^. In contrast, in the “bridge” model, H2Bub directly recruits factors for H3K4 and H3K79 methylation. However, because H2Bub does not affect the association of Set1 and Dot1 with chromatin^55,56^, H2Bub is required for the recruitment of other regulatory factor(s) to mediate crosstalk. Furthermore, H2Bub facilitates the association of Swd2 with the COMPASS complex (complex of proteins associated with Set1) and triggers the recruitment of proteasomal ATPases Rpt4 and Rpt6 to chromatin, which further mediates H3 methylation at K4 and K79^47,57^. Although H2Bub is not required for the chromatin binding of Spp1^58^, another subunit of COMPASS, the H2Bub-stimulated ubiquitination of Swd2subsequently recruits Spp1 for efficient H3K4 methylation^59^. Additionally, the presence of H2Bub at the nucleosome repositions hDot1L (Dot1 in *S. cerevisiae*) on the nucleosomal surface, resulting in its positioning at the catalytic site of the enzyme by binding to K79 at H3^60^.

On the basis of the above facts, diverse factors can affect modifications of the histone tail. For instance, mutations in the H2AN terminus significantly decrease both H2Bub and H3K4 methylation without affecting the association of the modifying enzymes Rad6/Bre1 and COMPASS with chromatin^61^. In addition, H3R2 methylation abrogates H3K4me3 via the inhibition of Spp1 binding^62,63^, and this pathway is evolutionarily conserved, as H3R2 methylation by PRMT6 mutually antagonizes H3K4 methylation by the SET1/MLL (mixed lineage leukemia) complex in mammals^64^. In another trans-tail pathway, PRMT7-mediated H4R3 methylation interferes with the binding of the PHD finger, thereby recognizing a methylated lysine in MLL3/4 during cellular differentiation^65,66^.

### Trans-tail regulation and silencing

The role of the trans-tail pathway was first postulated to be a regulator of transcriptional silencing because H2BK123R mutation impairs the repression of the *URA3* reporter gene, located in the left-end telomere of chromosome VII^67^. In *S. cerevisiae*, there are three heterochromatin loci, subtelomeric, rDNA (rRNA-encoding DNA), and mating-type regions^68^, in which a characteristic pattern of histone modifications has been observed^67,69,70^. In particular, the Sir2 HDAC-mediated regulation of histone acetylation levels governs locus-specific chromatin condensation^68^. This Sir2 association is in turn tightly regulated by the trans-tail pathway^70^. Therefore, the loss of enzymes for H2Bub or H3K4/K79 methylation disrupts the silencing of the *URA3* reporter gene at all heterochromatin loci^70–72^, whereas Ubp8 and Jhd2 H3K4 demethylases have an anti-silencing function in rDNA regions^70,73^. In addition, the modification-mediated control of Sir2 recruitment further affects cellular aging by maintaining intact telomeric chromatin^70,74^.

### Trans-tail regulation and the Set3 pathway

The trans-tail pathway involves not only heterochromatic silencing but also transcriptional regulation, and its cellular function in the transcription system has been intensively studied. H2Bub-dependent H3K4 methylation exhibits an intrinsic gradient pattern, comprising me3 near promoters, me2 immediately downstream, and me1 in more-distal regions^75,76^, which is determined by the amount of time that Set1 is tethered to RNAPII during multiple rounds of transcription^77^. Therefore, such patterns of H3K4 methylation suggest that this modification is positively correlated with active transcription. However, the loss of Set1 causes only minor defects in the effect of this modification during gene expression. Furthermore, a genome-wide analysis showed that only 69 and 20 transcripts were significantly increased and decreased, respectively, upon the deletion of *SET1* or H3K4R mutation^78^. Therefore, it is proposed that the effects of this modification on transcription are more diverse and that it regulates several functions, including learning, memory, processing, and termination^79,80^

Although the function of H3K4me3 in transcription has been comparatively well studied, that of H3K4me2 remains unclear. However, Buratowski’s group showed that H3K4me2 has distinct effects on the transcription cycle a decade ago^13^. The expression of Set1 lacking the RRM (RNA Recognition Motif) domain, which eliminates H3K4me3 but has no effect on H3K4me2^72^, causes no apparent increase in histone acetylation in 5′ transcribed regions, suggesting that H3K4me2 is sufficient to suppress histone acetylation in the 5′ ORF region^13^. Furthermore, among several candidate proteins, including proteins with the PHD domain that binds methylated H3K4 in vitro^81^, the Set3 protein preferentially binds H3K4me2 peptides. Subsequently, H3K4me2 influences the association of the Set3 complex, including two active HDAC subunits, Hos2 and Hst1, with chromatin^13^. The loss of such subunits and additional accessory proteins, Sif2 and Snt1, in the Set3 complex increases histone acetylation at 5′ ORF loci^13^. Therefore, the Set3 complex-mediated deacetylation of histones in 5′ ORFs represses the cryptic initiation of both sense and antisense transcription in gene bodies^14^. Taken together, these findings indicate that H2Bub and the subsequent H3K4me2-driven histone deacetylation process maintain transcriptional fidelity by suppressing spurious transcriptional initiation.

### Trans-tail regulation and histone sumoylation

Recent findings revealed that histone sumoylation is also closely related to this trans-tail regulation. Histones are an evolutionarily conserved target of small ubiquitin-like modifier (SUMO) modification, which is sequentially carried out by the following enzymes: heterodimeric Aos1/Uba2 (SAE1/SAE2 in mammals), SUMO-activating enzyme (E1), Ubc9 SUMO-conjugating enzyme (E2), and several SUMO ligases (E3s)^12,82^. Since the first reports of human histone-SUMO conjugates in 2003^83^, several sumoylation sites on four core histones and histone variants have been discovered in human cells and *S. cerevisiae*^12^. In the case of histone variants, the sumoylation of the H3 variant Cse4 mediates its proper localization at the centromere^84–86^, and the repair of DNA double-strand breaks is affected by H2A.Z sumoylation^87^. Until recently, the effect of SUMO on core histones was assumed to be transcriptional repression via the inhibition of, or competition with, gene activation markers such as monoubiquitination and acetylation on histones or the recruitment of their enzymes^12^.This type of modification-dependent control is not simple, and it contributes to the remarkably complex transcription program.

**Fig. 3:**
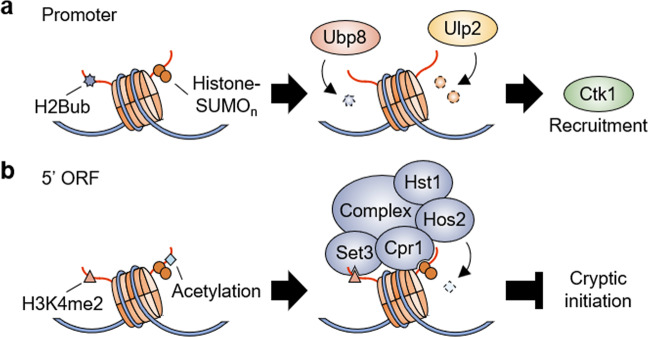
Dual functions of histone sumoylation in transcription. **a** In the promoter region, the elimination of ubiquitin and SUMO from histones by Ubp8 and Ulp2, respectively, recruits Ctk1 to chromatin to facilitate the transition between transcriptional initiation and elongation. **b** In the 5′ ORF region, H3K4me2 and histone (poly)sumoylation individually provide binding platforms for Set3 and Cpr1 in the Set3 complex, including two HDAC enzymes, Hst1 and Hos2, to suppress internal cryptic initiation.

The correlation between the trans-tail pathway and histone sumoylation was incidentally discovered in a functional study of the Ulp2 protease^88^, which efficiently disassembles poly-SUMO chains on proteins and acts as an essential regulator of cell homeostasis in *S. cerevisiae*^89–92^. Ulp2 is preferentially associated with constitutive genes, and its loss impedes efficient gene expression via a defect in RNAPII recruitment^88^. Genetic interactions of *ULP2* with genes encoding H2Bub enzymes, *RAD6* and *BRE1*, were first discovered in synthetic lethal screening assays, implying that these genes are involved in similar pathways^88^. Furthermore, H2Bub is required for the histone sumoylation and chromatin localization of Ulp2, allowing subsequent histone desumoylation steps during transcription^88^. Furthermore, persistent polySUMO conjugation to H2B or Ulp2 loss blocks Ctk1 nucleosome binding, thus limiting CTDS2 phosphorylation and efficient transcriptional elongation^88^. Such defects in Ctk1 recruitment are similarly observed in cells lacking Ubp8^43^, suggesting that both ubiquitin and SUMO conjugation to histones serve to modulate the level of CTDS2 phosphorylation required for the efficient transition between transcriptional initiation and elongation steps (Fig. 3a).

Similar to the effect of H2Bub on histone sumoylation, H3K4me2, but not me3, is a prerequisite step for histone sumoylation to occur, and this pathway is unidirectional and not bidirectional^93^. Additionally, a histone H2B mutation causing defective H2B sumoylation impairs the association of two subunits of the Set3 complex, Set3 and Hos2, with target genes. This process results in hyper-histone acetylation, and shows strong sensitivity to 6-azauracil, which is a general indicator employed for evaluating transcriptional elongation^94^, at 34 °C^93^. Another subunit Cpr1 of the Set3 complex recognizes SUMO-conjugated histones via its SIM (SUMO-interacting motif) and promotes Set3 complex loading onto nucleosomes^93^. Notably, such H2B mutation impedes the recruitment of the Set3 complex to ncRNA (noncoding RNA) genes as well as protein-coding genes, which greatly increases the transcription of ncRNAs from internal sites within ORFs^93^. Therefore, an elaborate histone modification network involving the consecutive ubiquitination, methylation, sumoylation and deacetylation of histones promotes transcriptional elongation by suppressing cryptic intragenic initiation (Fig. 3b).

## Concluding remarks

The effect of H2Bub on H3K4 and H3K79 methylation itself has been well characterized, and this trans-tail regulation pathway is clearly linked with chromatin dynamics and diverse nuclear functions^36^. However, the ultimate role of this crosstalk pathway in transcription has remained unclear until recently. Here, we have briefly reported that the transcriptional mechanism is elaborately regulated by the H2Bub-originated regulation pathway. In an early transcription stage, the CTDS5-phosphorylated form of RNAPII and the Paf1 complex are both required for Rad6 and Bre1-mediated H2Bub^38,39^, resulting in two sequential histone modifications, Set1-mediated H3K4 methylation and histone sumoylation by Ubc9 and E3 ligase(s)^46,47,88,93^. However, both H2Bub and histone sumoylation act as barriers to Ctk1-dependent CTDS2 phosphorylation, which then favors the removal of ubiquitin and SUMO proteins from histones catalyzed by Ubp8 and Ulp2, respectively. This process subsequently facilitates CTDS2 phosphorylation by Ctk1^43,88,93^. During transcriptional elongation, H2Bub and histone sumoylation cycles are repeated^43,88,93^, while an H3K4 methylation gradient in which H3K4me3 in the promoter and H3K4me2 occurs in the 5′ ORF is gradually established^76,77^. In further steps, the Set3 and Cpr1 subunits of the Set3 HDAC complex recognize H3K4me2 and histone sumoylation, respectively, which then facilitates the deacetylation of histones in 5′ ORF regions to prevent cryptic internal initiation^13,88,93^.

It remains unclear whether the trans-tail pathway-mediated suppression of spurious transcription is evolutionarily conserved in mammals. However, the mammalian ortholog of Set3 is MLL5, as suggested by their sequence similarities. Both proteins also lack intrinsic methyltransferase activity^95^. Furthermore, MLL5 is a component of the NCoR–SMRT complex, which acts as a corepressor of hormone receptors and recruits HDAC to regulate gene expression^96^. Moreover, despite the functional correlation between the Set3 complex and NCoR–SMRT, the role of MLL5 in the inhibition of cryptic transcription has not yet been determined. Although Dot1-dependent H3K79 methylation is involved in active transcription and genome stability^97^, no evidence related to cryptic initiation has been reported. In addition, the effect of histone sumoylation on the Set3 complex has been studied only in *S. cerevisiae*^93^, and functional studies on histone sumoylation in higher eukaryotes are required to understand its complex mechanism.

On the basis of the above findings, the misregulation of H2Bub or histone methylation has been associated with several human diseases, including cancers and neurodegenerative disorders^98,99^. Therefore, although there are no available reports of the effects of cryptic transcription inhibited by trans-tail regulation in human diseases, many diseases are known to be closely correlated with aberrant expression of ncRNAs, which are key factors in gene expression control, genome stability, and chromatin dynamics^100^. Hence, the better characterization of how epigenetic regulation modulates the monitoring mechanisms of spurious transcription may be a promising avenue for future research to develop new therapies for various disorders.
